# Progress of ADAM17 in Fibrosis-Related Diseases

**DOI:** 10.1155/mi/9999723

**Published:** 2025-02-26

**Authors:** Suyan Yan, Yaqi Zhao, Yuyu Yang, Baocheng Liu, Wei Xu, Zhenzhen Ma, Qingrui Yang

**Affiliations:** ^1^Department of Rheumatology and Immunology, Shandong Provincial Hospital Affiliated to Shandong First Medical University, Jinan 250021, Shandong, China; ^2^Department of Rheumatology and Immunology, Shandong Provincial Hospital, Cheeloo College of Medicine, Shandong University, Jinan 250021, Shandong, China; ^3^UCL School of Pharmacy, University College London, London, UK; ^4^Shandong University of Traditional Chinese Medicine, Jinan 250021, Shandong, China

**Keywords:** ADAM17, EGFR, fibrosis

## Abstract

Fibrosis leads to structural damage and functional decline and is characterized by an accumulation of fibrous connective tissue and a reduction in parenchymal cells. Because of its extremely poor prognosis, organ fibrosis poses a significant economic burden. In order to prevent and treat fibrosis more effectively, potential mechanisms need to be investigated. A disintegrin and metalloprotease 17 (ADAM17) is a membrane-bound protein. It regulates intracellular signaling and membrane protein degradation. Fibrosis mediated by ADAM17 has been identified as an important contributor, although the specific relationship between its multiple regulatory functions and the pathogenesis is unclear. This article describes ADAM17 activation, function, and regulation, as well as the role of ADAM17 mediated fibrosis injury in kidney, liver, heart, lung, skin, endometrium, and retina. To develop new therapeutic approaches based on ADAM17 related signal pathways.

**Summary**

• This review focuses on A disintegrin and metalloprotease 17 (ADAM17) components and its role in fibrosis and presents research progress.
• We describe the specific mechanisms by which ADAM17 induces fibrosis in different organs, including the kidney, liver, heart, lung, skin, endometrium, and retinal.
• We explore the possible factors through which ADAM17 contributes to fibrosis via inflammatory factors and cytokines in acute and chronic diseases are explored.
• We outline the most studied mechanisms underlying ADAM17-induced fibrosis and their potential implications.

## 1. Introduction

**Figure 1:**
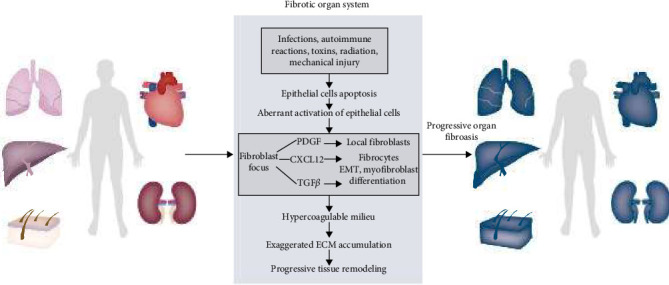
The general fibrotic process. CXCL12, chemokine (C-X-C motif) ligand 12; ECM, extracellular matrix; EMT, epithelial-to-mesenchymal transition; PDGF: platelet-derived growth factor; TGF-*β*: transforming growth factor-*β*. Drawing using Adobe illustrator software.

In most systemic disease processes, fibrosis is a process of scar repair. In response to specific cytokines and oxidative stress, replacement of native tissue with dense connective tissue due to homeostatic imbalances, leads to normal tissue becoming dysfunctional, organ dysfunction, and significant morbidity and mortality [1, 2] (Figure 1). A disintegrin and metalloprotease 17 (ADAM17), also known as tumor necrosis factor-*α* (TNF-*α*) transform enzyme, is a membrane-associated enzyme that breaks down cell surface proteins [3–5]. Through its shedding enzyme function, ADAM17 is a regulator of cell propagation, growth, inflammation, and other cellular processes [6]. Dysregulation of ADAM17 has been strongly implicated in a variety of pathological conditions including asthma, cancer, arthritis, and fibrosis [7–9]. The purpose of this comprehensive review is to provide insight into the structure, activation, and regulation of ADAM17 during fibrosis development and to discuss how ADAM17-induced long-term inflammation and advanced fibrosis exacerbate organ failure. The family of ADAMs and their functions in the absence of pathology are shown in Table S1.

## 2. Biological Characteristics of ADAM17

**Figure 2:**
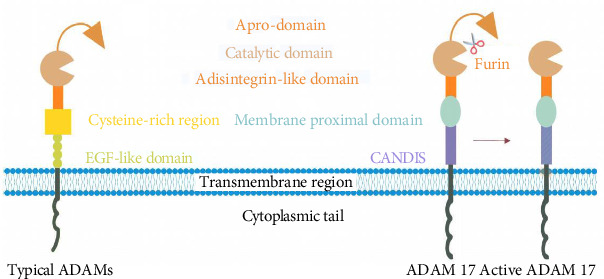
The structure of classical ADAMs and ADAM17. The structure of classical ADAMs consists of a pro-domain, catalytic domain, a disintegrin-like domain, a cysteine-rich region, an EGF-like domain, a transmembrane region, and a cytoplasmic tail. ADAM17 includes a pro-domain, catalytic domain, a disintegrin-like domain, a membrane proximal domain, CANDIS domain, transmembrane region, and a cytoplasmic tail. ADAM17: A disintegrin and metalloprotease 17; EGF-like domain: epidermal growth factor-like domain. Drawing using Adobe illustrator software.

An ADAM is a family of type I transmembrane and secreted metalloendopeptidases that act by cleaving ectodomains of membrane anchored growth factors, cytokines, and receptors [10, 11]. As of today, 22 ADAMs have been recognized in the human genome. ADAMs are generally composed of a number of characteristic protein domains: an N-terminal signal sequence surrounded by a prodomain, a catalytic domain, a disintegrin domain, a cysteine-rich region, an epidermal growth factor (EGF)-like domain, a transmembrane region, and a cytoplasmic tail [12–14]. As opposed to the typical ADAMs, ADAM17 does not contain an EGF-like domain, instead, it consists of a membrane-proximal domain. A membrane proximal domain is followed by a brief stalk sequence that is highly conservation across the animal kingdom and is known as the CANDIS domain [6, 15, 16]. ADAM17 matures by furin-dependent processing at either a canonical proprotein convertase (PC) cleavage site at the prodomain/catalytic domain interface [17] or an upstream PC cleavage site [18] (Figure 2).

The N-terminus of ADAM17 includes signaling sequences that direct the protein to the secretory pathway [12]. ADAM17′s prodomain is essential for intracellular transport and functions as an intramolecular chaperone to ensure proper protein folding [19, 20]. Additionally, it prevents the catalytic activity of the enzyme via a cysteine switch mechanism [21] in which cysteine residues in the pro-domain coordinate zinc ions in the catalytic site, inhibiting catalysis [22]. In terms of its catalytic domain, ADAM17 shares some similarities with snake venom metalloproteinases [23, 24] and is relatively resistant to tissue inhibitors of metalloproteinase-1 [25]. This disintegrin-like domain is critical for interacting with integrins, mediating adhesion between neighboring cells, activating various receptors, and initiating various cell signaling pathways [26]. Additional, recombinant expression of the ADAM17 disintegrin-like domain impairs fibroblast-cancer cell interaction [27]. Protein disulfide isomerase interacts with the membraneproximal domain to participate in dimerization and substrate recognition, resulting in structural changes between the activated open state and the inactive closed conformation [28, 29]. A key substrate of ADAM17, the interleukin-6 receptor (IL-6R), is mediated by CANDIS, whose substrate binding properties are modulated by its membrane proximal domain [16, 30]. The distinct compositions of the cytoplasmic tails are critical for their distinct roles in metalloproteinase activity, intracellular signaling, and subcellular localization. As a consequence, ADAM17 plays a significant role in physiopathological processes throughout the body.

## 3. Role of ADAM17 in Fibrosis

### 3.1. ADAM17 and Renal Fibrosis

As shown by RNA in situ hybridization in human normal kidneys, ADAM17 gene expression is highest in the distal tubules. Conversely, ADAM17 gene expression observed in glomerular endothelium, glomerular mesangium, peritubular capillaries, and proximal tubules [31]. The glomerular parietal epithelium and podocytes of patients with chronic renal disease express high levels of ADAM17 mRNA. De novo expression has also been reported in the glomerular endothelium, mesangium, peritubular capillaries, and proximal tubules in kidney disease [31, 32].

Renal fibrosis is marked by the overproduction and enrichment of extracellular matrix (ECM) proteins in the kidney, leading to parenchymal scarring, renal failure, and eventually end stage renal disease (ESRD) [33, 34]. An essential role is performed by the EGF receptor (EGFR), a member of the ErbB family of tyrosine kinase receptors. Acute kidney injury (AKI) has been shown by genetic knock out and pharmacological inhibition studies to be protected by early activation of the EGFR pathway, while sustained EGFR signaling leads to renal injury and fibrosis in experimental AKI [35–37]. A study by Liu et al. [36] showed that unilateral ureteral obstruction (UUO) induced renal fibrosis significantly decreased smooth muscle actin expression, a hallmark of activated fibroblasts, and reduced collagen and fibronectin accumulation in the kidney by pharmacological inhibition or genetic reduction of EGFR activity [38]. To activate EGFR, membrane-bound precursors must be converted into soluble ligands by shedding enzymes. Recombinant EGFR ligands, for example, amphiregulin (AREG), heparin-binding EGF (HB-EGF), epiregulin (EREG), and transforming growth factor-*α* (TGF-*α*), are predominantly shed by ADAM17 [39]. After total body irradiation, targeted AREG mitigated the development of renal fibrosis. An intravenous injection of SAMiRNA targeting mouse AREG mRNA decreased radiation induced collagen accumulation in the renal cortex and medulla [40]. AREG has been demonstrated to upregulate ADAM17 in vitro [39]. In human proximal tubular epithelial cells, quercetin significantly improved TGF-*β*1 (TGF-*β*1) induced epithelial-to-mesenchymal transition (EMT), which was accompanied by increased AREG expression. It has also been shown that quercetin inhibits AREG binding to EGFR, suggesting that it alleviates fibrosis by inhibiting AREG/EGFR signaling [41]. A renal fibrosis model was established in mice induced by angiotensinogen II (Ang II) and its sheddase, metalloprotease-17. Targeted deletion of TGF-*α* or inhibiting its cleavage with a specific metalloprotease-17 inhibitor significantly reduced Ang II-induced EGFR phosphorylation and renal injury, indicating the relevance of the ADAM17/TGF-*α*/EGFR pathway in Ang II-induced renal injury [42]. TNF-*α* and HB-EGF, both ADAM17 substrate, are associated with renal damage in lupus nephritis by activation of the EGFR signal pathway and by causing renal inflammation [43], indicating a role for EGFR ligands or EGFR in the pathogenesis of renal fibrous formation.

In the classical interleukin-6 (IL-6) signaling pathway, IL-6 binding to membrane IL-6Rs triggers signaling through glycoprotein 130 (gp130) as a signal transducer [44, 45]. Nevertheless, this signaling pathway depends on the presence of both IL-6R and gp130 on the same cell membrane. Experimental crescentic glomerulonephritis appears to be benefited by classical IL-6 signaling [46, 47]. However, IL-6 trans-signaling is thought to be involved in the progression of kidney disease. IL-6 trans-signaling involves ADAM17, which converts membrane-bound IL-6R to a soluble receptor that forms a soluble complex that stimulates cells with only gp130 on their membranes (e.g., renal endothelial, epithelial, or mesangial cells). Recombinant soluble gp130 extracellular structural domain coupled to the Fc portion of IgG prevents renal fibrosis by suppressing gp130/signal transducers and activators of transcription (STAT) 3 in various mouse models [48]. Thus, inhibition of ADAM17 may reduce IL-6 trans pathway-mediated renal injury, while preserving classical nephroprotective IL-6 signaling.

Injury increases the expression of ADAM17 and its substrates, enhancing ADAM17 activity on the cell surface and increasing the release of soluble TNF-*α*, which activates TNF receptor-1 and receptor-2 (TNFR1/2). The TNF-*α*/TNFR1/2 pathway leads to tissue necrosis and proinflammatory cytokines, resulting in renal fibrosis after kidney injury [39]. In another study, a mouse model of type 1 diabetes was used to examine the role of endothelial ADAM17 (eADAM17) and proximal tubular ADAM17 (tADAM17) deficiencies in the regulation of the renin–angiotensin system (RAS), renal inflammation, and fibrosis. In addition, eADAM17 deficiency reduced renal fibrosis and inflammation, whereas tADAM17 deletion reduced podocyte loss, attenuated the RAS and decreased macrophage invasion, smooth muscle actin, and collagen accumulation [49]. ADAM17 promotes angiotensin-converting enzyme 2 (ACE2) shedding to induce thylakoid matrix expansion, collagen deposition, and progression of diabetic nephropathy [50]. Inhibition of ADAM17 reduced renal fibrosis in mice with Ang II-induced kidney disease [51]. These findings indicate that ADAM17 may regulate downstream signaling pathways by activating the RAS through ACE2 cleavage.

### 3.2. ADAM17 and Liver Fibrosis

As a result of a variety of chronic liver diseases, liver fibrosis can develop. This condition is characterized by long-term parenchymal injury or chronic inflammation, which stimulates the transdifferentiation of hepatic stellate cells into stromal secretory myofibroblasts.

A heterogeneous population of proliferating, migrating, and profibrotic cells, liver myofibroblasts play a major role in promoting liver fibrosis [52]. Physiological and pathological changes in the liver can be influenced by ADAM17 under normal or abnormal conditions. ADAM17-deficient mice are lethal perinatally and show development defects related to EGFR signaling in the eye, hair, and skin [53]. ADAM17 h-KO mice expressing hepatocyte-specific knockouts of ADAM17 were significantly attenuated by partial hepatectomy following two-thirds partial hepatectomy, indicates that ADAM17 is the primary sheddase that produces these factors [54]. The TNFR1 receptor regulates liver regeneration and synergizes with the EGFR signaling pathway within the liver cells, initiating DNA replication cascades [55]. Additionally, ADAM10- and ADAM17-deficient mice in a partial hepatectomy model showed reduced protein kinase B phosphorylation and decreased release of EGFR-activating factors, indicating that synchronous ablation of ADAM10 and ADAM17 results in inhibited EGFR signaling [56].

C-mer tyrosine kinase (MerTK) is a member of the Tyro-Axl-MerTK family of proteins. It is expressed at high levels on macrophages and has various ligands, including Gas6 and protein S [57, 58]. Human genetic studies have shown that two single-nucleotide intronic polymorphisms in MerTK, rs4374383 A and rs6726639 A, are linked to reduced hepatic expression of MerTK and decreased risk of liver fibrosis [59, 60]. Interestingly, ADAM17 is capable of cleaving MerTK into soluble Mer (s-Mer) [61, 62]. In nonalcoholic steatohepatitis, limiting ADAM17 activity increases MerTK expression in macrophages, which further induces TGF-*β*1 production, leading to the activation of hepatic stellate cells by TGF-*β*1 to encourage liver fibrosis [63].

ADAM17 maturation is triggered by cofactors such as inactive rhomboid protein 2 (iRhom2), an ineffective member of the rhomboid protease family that is encoded by the Rhbdf2 gene [64–67]. iRhom2 and ADAM17 are both found in the endoplasmic reticulum, where iRhom2 facilitates the translocation of ADAM17 to the plasma membrane [64]. ADAM17 activity is enhanced following deletion of the cytoplasmic tail, leading to increased TNFR shedding and decreased apoptosis following TNF-*α* exposure [65]. Phosphorylation of the cytoplasmic tail of iRhom2 leads to dissociation of ADAM17, accompanied by an enhancement of its catalytic activity [68, 69]. The liver of iRhom1 and 2 double knockout mice lacked mature ADAM17 expression. Similarly, iRhom2 knockout mice showed no reduction in mature ADAM17 expression in the liver [70]. It has been shown that iRhom2 deletion reduces the shedding of ADAM17 substrates (such as TNF-*α*, L-selectin, HB-EGF, TNFR1, TNFR2, and AREG) [65, 71, 72]. iRhom2 is a key regulator of multiple aspects of ADAM17 biology, including its maturation and catalytic activity, as these studies demonstrate. In a bile duct ligation (BDL) mouse model of liver fibrosis, Rhbdf2-/-BDL mice were found to have reduced ADAM17 activation and TNFR shedding following BDL, suggesting an increase in unbound TNFR on the surface of hepatic stellate cells or hepatocytes, promoting TNF-*α* binding to TNFR and driving hepatic stellate cell growth and liver fibrosis [73]. Although iRhom2 deficiency decreases TNF-*α* release, it does not completely prevent it [74]. In addition, unshed membrane-bound TNF-*α* can still bind and activate both TNFR1 and TNFR2, albeit with varying efficiencies [75, 76]. The result, ADAM17 contributes to a variety of liver diseases and fibrosis through substrate cleavage, but the specific mechanisms remain a mystery.

### 3.3. ADAM17 and Myocardial Fibrosis

Myocardial fibrosis is a shared pathophysiological process in numerous cardiac diseases, including heart attack, hypertension, and heart failures, and is marked by excessive deposition of ECM proteins [77]. This process is originally intended to safeguard cardiac function by the repair, replacement, and strengthening of heavily or permanently damaged tissue. Over-deposition of ECM decreases ventricular compliance and cardiomyocyte contractile function, resulting in heart failure and increased mortality [78].

ADAM17 activates AREG through proteolytic cleavage. The AREG protein is either consolidated as a type I transmembrane protein, which may be involved in signaling in bordering cells, or released as soluble AREG after protein hydrolysis, acting as an autocrine or as a paracrine factor [79, 80]. AREG soluble expression is widespread on cardiomyocytes and fibroblasts, acting as a ligand for EGFR [79]. EGFR activation in the heart triggers important intracellular signaling pathways that control fibroblast proliferation, migration, and collagen synthesis. In chronic stress states marked by sustained elevation of soluble AREG, however, prolonged EGFR activation enhances cardiac fibroblast activation, proliferation, and myofibroblast differentiation mediated by the Janus kinase (JAK)/STAT pathway and promotes cell migration and collagen synthesis, contributing to cardiac fibrosis [79]. Increased levels of TNF-*α* and IL-6 secreted by macrophages and T and B lymphocytes during chronic inflammation also contribute to the exacerbation of cardiac fibrosis, whereas upregulation of ADAM17 enhances the secretion of these cytokines by innate and adaptive immune cells through either direct or indirect cascades, thereby, contributing to maladaptive interstitial fibrosis [81, 82].

Ang II and its generating enzyme ACE, the main axis of the renin–angiotensin–aldosterone system (RAAS), have long been linked to the incidence and severity of cardiovascular disease [83]. Vasoconstriction is caused by Ang II [84], proliferation by Ang II [85], inflammation by Ang II [86], and coagulation by Ang II [87]. There is increasing support that another RAAS pathway has a protective effect on the heart [88, 89]. A homolog of ACE, ACE2, which is cleaved by ADAM17, reduces myocardial fibrosis, hypertrophy, and apoptosis by binding to the MAS receptor (MASR) [90]. In spite of this, experimental studies have shown conflicting results regarding ADAM17′s role in cardiomyopathy. The expression of ADAM17 is elevated in the myocardium of patients with dilated cardiomyopathy and hypertrophic obstructive cardiomyopathy in animal models [91]. In Ang II-induced hypertensive mice and in spontaneously hypertensive rats, inhibition of ADAM17 with small interference RNA inhibited myocardial hypertrophy and fibrosis [92]. ADAM17 appears to be a double-edged sword, potentially useful only in circumstances where the RAS is highly activated.

### 3.4. ADAM17 and Pulmonary Fibrosis

As a consequence of excessive deposition of ECM proteins by active lung fibroblasts and myofibroblasts, pulmonary fibrosis leads to decreased gas exchange and reduced lung function [93]. According to a clinical study, serum ADAM17 expression was noteworthy higher in patients with inflammatory myopathies than in healthy individuals. In addition, patients who combined with idiopathic pulmonary fibrosis (IPF) had higher ADAM17 expression than patients with inflammatory myopathies alone [94], highlighting elevated ADAM17 expression in patients with inflammatory myopathies, particularly IPF. An additional clinical study confirmed that ADAM17 expression was higher in IPF patients and connective tissue diseases as well as interstitial lung disease than in healthy controls, with patients with acute exacerbations of IPF showing significantly higher ADAM17 expression than those with stable IPF; these findings suggest that ADAM17 plays a significant role in the progression of pulmonary fibrosis [95]. ADAM17 expression is widespread throughout the lung tissue [96]. TGF-*β* induces increased expression of connective tissue growth factor in human lung epithelial cells through the signal transduction pathway of extracellular signal-regulated kinase (ERK)/ADAM17/ribosomal S6 kinases 1 (RSK1)/CCAAT/enhancer-binding protein *β* (C/EBP*β*), thereby, promoting pulmonary EMT and increasing fibronectin levels. Evidence also suggests that the ADAM17–EGFR–ERK pathway contributes to lung fibrosis [97, 98]. Therefore, ADAM17 has an important role in lung fibrosis and may be a therapeutic target.

### 3.5. ADAM17 and Dermal Fibrosis

Several inflammatory or autoimmune skin diseases can cause dermal fibrosis, which is defined by an excessive accumulation of ECM constituents in the dermis [99]. The Notch signaling pathway has been implicated in fibrosis [100]. Notch has been reported to be activated in patients with either localized scleroderma or diffuse cutaneous systemic sclerosis (SSc) [101]. In mammals, the Notch family is composed of four transmembrane receptors (Notch-1 to Notch-4) and five ligands (Jagged1, Jagged2, Delta1, Delta3, and Delta4). At all stages of the development, Notch signaling is implicated in cell proliferation, survival, apoptosis, and differentiation [102–104]. A ligand must bind to the Notch ectodomain to trigger its shedding by ADAM-10 or ADAM-17 during Notch signaling [105, 106]. Thus, ADAM17-mediated regulation of dermal fibrosis is via Notch signaling. Furthermore, ADAM17–EGFR promotes dermal fibrosis in SSc mice in conjunction with increased inflammation caused by phorbol 12-myristate 13-acetate (PMA) in skin fibroblasts [107].

### 3.6. ADAM17 and Endometrial Fibrosis

The existence of endometrial tissue outside the uterine cavity is characteristic of endometriosis, a benign gynecological inflammatory disease [108]. Research has shown that oxidative stress plays an essential role in the development and course of endometriosis [109, 110]. Endometriosis is also characterized by fibrosis, which is seen in ectopic lesions and is strongly associated with the most serious forms of the disease [111]. ADAM17 expression is elevated during inflammatory processes, especially in the presence of reactive oxygen species (ROS) [112, 113]. There was a significantly positive correlation between the levels of ADAM17 proteinase and advanced oxidation protein products in the peritoneal fluid of patients with endometriosis [114]. ADAM17 has also been shown to play a major role in the proteolysis of NICD (from the Notch-1 receptor) following ligand binding in in vitro experiments [115]. There are two consecutive cleavages of the active Notch receptor that release the NICD following activation of the Notch receptor. A NICD translocates to the nucleus and is implicated in the transcriptional regulation of nuclear target genes leading to fibrosis [101]. According to the above studies, ADAM17/Notch may play a role in the process of endometrial fibrosis.

### 3.7. ADAM17 and Retinal Fibrosis

**Figure 3:**
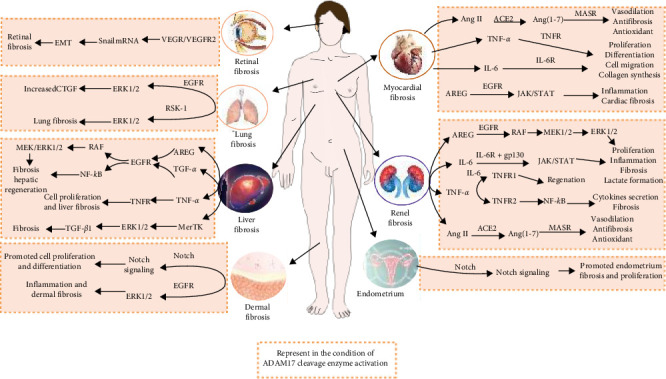
The organ fibrotic process in the condition of ADAM17 activation. ACE2, angiotensin-converting enzyme 2; ADAM17, a disintegrin and metalloprotease 17; Ang (1-7), angiotensinogen (1-7); Ang II, angiotensinogen II; AREG, amphiregulin; CTGF, connective tissue growth factor; EGFR, epidermal growth factor receptor; ERK, extracellular signal-regulated kinase; gp130, glycoprotein 130; JAK, Janus kinase; IL-6, interleukin-6; IL-6R, IL-6 receptor; MASR, MAS receptor; MEK, mitogen-activated protein kinase; MerTK, C-mer tyrosine kinase; RSK-1, ribosomal S6 kinases-1; STAT, signal transducers and activators of transcription; TGF-*α*, transforming growth factor-*α*; TGF-*β*1, transforming growth factor-*β*1; TNF-*α*, tumor necrosis factor-*α*; TNFR, TNF receptor; TNFR1/2, TNF receptor-1/2. Drawing using Adobe illustrator software.

Vascular endothelial growth factor (VEGF) is critical for tumor neovascularisation, the formation of blood vessels within a tumor, which facilitates cancer cell survival, local tumor growth, and the formation of distant metastases [116]. VEGF binds to two different receptors, VEGF receptor 1 (VEGFR1) and VEGF receptor 2 (VEGFR2), with VEGFR2 thought to be the most important signaling receptor for endothelial cell membrane permeability, specification, and proliferation [117–119]. Increased levels of VEGF have been found in vitreous samples from patients with retinal neovascular disease, suggesting a role for VEGF in retinal disease [120]. Retinal pigment epithelial (RPE) cells, located between the choroid and the retina, play a critical role in the methodogenesis of age-related macular degeneration (AMD) [121]. RPE cells also seize a variety of cytokines, chemokines, and growth factors. These include IL-6, IL-11, chemokine (C-X-C motif) ligand 9, platelet-derived growth factor (PDGF), TGF-*β*, and VEGF [122]. TGF-*β* and VEGF have been shown to significantly regulate Snail mRNA, which is associated with EMT. VEGF promotes VEGFR2 shedding via ADAM17, thereby, promoting the EMT process [123]. Evidence that ADAM17 is in relation to the pathogenesis of retinal fibrosis. A previous study indicated that inhibition of VEGF-mediated EMT signaling by cotreatment with vandetanib and an ADAM inhibitor is a novel and potentially promising therapeutic intervention for retinal pathologies [124].  Figure 3 summarizes the organ fibrotic process under ADAM17 activation.

## 4. Limitations

Our review of ADAM17 pathogenesis and fibrosis-related diseases revealed that the relevant mechanisms have not yet been fully explained and the conclusions are not entirely consistent. Thus, ADAM17 is still a target for therapeutic intervention in fibrosis-related diseases and much research is needed before it can be proven efficacious.

## 5. Outlook

As discussed, ADAM17 is primarily studied as a disease-promoting cleavage protein, which is widely expressed in somatic cells and cleaves various substrates (including TNF-*α*, HB-EGF, AREG, IL-6R, and Notch) to promote disease onset and fibrosis development; it also plays protective roles, for instance, ADAM17 cleaves MerTK into s-Mer, which, through the ERK/TGF*β*1 pathway, can be profibrotic. The inhibition of MerTK cleavage by ADAM17 contributes to the development of fibrosis. Similarly, ADAM17 cleaves ACE2 in order to convert Ang II into Ang II-VII, which exerts a protective effect against fibrosis. Therefore, ADAM17′s role depends on the membrane proteins it cleaves.

This review focuses mainly on the profibrotic effect of ADAM17. There are currently no clinically available inhibitors of ADAM17, making it a potentially effective antifibrosis strategy. Previously, we were aware of two ADAM17 inhibitors: DPC 333 (BMS-561392) [125] and TMI-005 (apratastat) [126]. However, both of these agents have some inhibitory effect on different ADAMs as well have not progressed past phase II trials due to toxicity or lack of efficacy. As reported by Xu et al. [127], a novel inhibitor of the Notch activating/cleaving enzyme ADAM17, ZLDI-8, inhibited NOTCH protein degradation, thereby, reducing the expression of pro-survival/anti-apoptosis and EMT-related proteins. But even with inhibitors specific to ADAM17 and not other metallases, the side effects would be significant. As more ADAM17 substrates are identified, there is increasing evidence that ADAM17 plays a role in almost all cellular functions. Its function as a major sheddase makes it essential for normal mammalian development and adult life. However, some studies have shown that despite the large number of substrates that have been assigned to ADAM17, it is notable that only a small number (e.g., IL-6R, EGFR ligands, and TNF-*α*) have been implicated in ADAM17 disease activity [128]. It is, therefore, likely that inhibitors targeting ADAM17 pathogenesis-related substrates will be a research focus in the future. Despite the fact that ADAM17 is a zinc-dependent metalloprotease with minimal sequence similarity to other ADAMs, most small molecules capable of inhibiting ADAM17 activity have failed in clinical trials. In addition, there are adverse side effects associated with other inhibitors of ADAM proteases, insufficient resorption and the formation of noxious metabolites [129 ]. For instance, TAPI-1 and GI254023X, which have been identified as potential ADAM17 inhibitors, have been observed to result in significant adverse effects in clinical settings. This is attributable to the presence of hydroxamate-based zinc binding groups, which facilitate their attachment to other zinc-dependent proteases [129, 130]. The sequestration of zinc (II) ions in zinc-dependent proteases is predominantly facilitated by the major functional groups of hydroxamate (CONHO^−^), carboxylate (COO^−^), thiolate (RS^−^), and phosphorus (PO_2_^−^). Among the compounds examined, hydroxamic acid followed by formylhydroxylamine exhibited the highest selectivity and potency for zinc binding. Consequently, the identification of non-zinc-binding compounds and the subsequent assessment of their farmacokinetics has the potential to enhance specificity and mitigate the adverse effects that are associated with ADAM17 inhibition. Nikfarjam et al. [131] employed bioinformatics techniques. Consequently, five drugs that have FDA approval, including both raltegravir and conivaptan, as well as paclitaxel, saquinavir, and venetoclax, were identified as having the capacity to inhibit the activity of the metalloenzyme ADAM17. However, these drugs were found to lack the necessary zinc-binding functional groups when assessed in testing. Further in silico analysis has demonstrated that raltegravir exhibits a favorable interaction with the active site and amino acids, in addition to the most advantageous kinetic properties when compared to other compounds. This suggests the potential for repurposing raltegravir as an antiviral agent to inhibit both serious inflammatory responses and tumourigenesis. This phenomenon can be attributed to ADAM17 dysfunction. In addition, targeting iRhom2, a binding protein specific to ADAM17, to inhibit ADAM17 activity is an emerging trend in ADAM17 inhibitor research. This is because iRhom2 is predominantly expressed in immune cells. Therefore, inhibitors would only affect the shedding processes on these cells. This may help to limit immune cell-mediated signaling via shed TNF-*α* in chronic inflammatory diseases without potentially affecting EGFR-mediated regeneration functions in nonimmune cells. However, a potential target could be the transmembrane helix (TMH) of ADAM17, which is a known interaction site with iRhoms. Here, disruption of the interaction site would destabilize the iRhom-ADAM17 complex, which could lead to a downregulation of ADAM17 activity [132].

Although it is still difficult and much remains to be explored and clarified, this should not dissuade us from trying to identify the cogwheels in the regulation mechanism of ADAM17 that could provide promising therapeutic approaches for the selective modification of this enzyme.

## Data Availability

The data that support the findings of this study are available from the corresponding author upon reasonable request.

## Nomenclature

ADAM17: A disintegrin and metalloprotease 17
TNF-*α*: Tumor necrosis factor-*α*
EGF: Epidermal growth factor
PC: Proprotein convertase
IL-6R: IL-6 receptor
CKD: Chronic kidney disease
ECM: Extracellular matrix
EGFR: Epidermal growth factor receptor
AKI: Acute kidney injury
UUO: Unilateral ureteral obstruction
AREG: Amphiregulin
HB-EGF: Heparin-binding epidermal growth factor
EREG: Epiregulin
TGF-*α*: Transforming growth factor-*α*
EMT: Epithelial-to-mesenchymal transition
TGF-*β*1: Transforming growth factor-*β*1
Ang II: Angiotensinogen II
Ang (1-7): Angiotensinogen (1-7)
IL-6: Interleukin-6
gp130: Glycoprotein 130
STAT: Signal transducers and activators of transcription
TNFR1/2: TNF receptor-1 and -2
eADAM17: Endothelial ADAM17
tADAM17: Tubular ADAM17
RAS: Renin–angiotensin system
ACE2: Angiotensin-converting enzyme 2
MASR: Mas receptor
sIL-6R: Soluble IL-6R
JAK: Janus kinase
ERK: Extracellular signal-regulated kinase
MEK: Mitogen-activated protein kinase kinase
MerTK: C-mer tyrosine kinase
s-Mer: Soluble Mer
iRhom2: Rhomboid protein 2
BDL: Bile duct ligation
RAAS: Renin–angiotensin–aldosterone system
IPF: Idiopathic pulmonary fibrosis
SSc: Systemic sclerosis
PMA: Phorbol 12-myristate 13-acetate
C/EBP*β*: Enhancer-binding protein *β*
RSK1: Ribosomal S6 kinases 1
CTGF: Connective tissue growth factor.

## Supporting Information

Supporting Information Table S1. Family of ADAMs and their functions in the absence of pathology. ADAM: a disintegrin and metalloprotease, CHL1: close homolog of L1, EGF: epidermal growth factor, APP: amyloid precursor protein, CNS: central nervous system, MGC: macrophage-derived giant cell, TNF: tumor necrosis factor, TGF: transforming growth factor, LGI1: anti-leucine-rich glioma-inactivated 1, FASL: FAS-ligand, CD40L: CD40-ligand.
