# Supplementary material for: Progress of ADAM17 in Fibrosis-Related Diseases
Source: Mediators Inflamm. 2025 Feb 26;2025:9999723. doi: 10.1155/mi/9999723 (PMC11986189; doi:10.1155/mi/9999723)
Supplement: Supporting Information — Table S1. Family of ADAMs and their functions in the absence of pathology. ADAM: a disintegrin and metalloprotease, CHL1: close homolog of L1, EGF: epidermal growth factor, APP: amyloid precursor protein, CNS: central nervous system, MGC: macrophage-derived giant cell, TNF: tumor necrosis factor, TGF: transforming growth factor, LGI1: anti-leucine-rich glioma-inactivated 1, FASL: FAS-ligand, CD40L: CD40-ligand. [file 9999723.f1.docx]

Supplementary Table 1. Family of ADAMs and their functions in the absence of pathology

| Human ADAM gene | physiologic processes | ADAM knockout mice | Suggest function |
| --- | --- | --- | --- |
| ADAM1 | Fertilization |  | Possibly involved in sperm-egg fusion |
| ADAM2 | Fertilization |  | Possibly involved in sperm-egg fusion |
| ADAM3 | Fertilization |  | Possibly involved in sperm-egg fusion |
| ADAM5 |  |  | Unknown |
| ADAM6 |  |  | Unknown |
| ADAM7 | Fertilization |  | Possibly involved in sperm-egg fusion |
| ADAM8 | Central nervous system function | shedding of the close homologue of L1 (CHL1) adhesion molecule was reduced in brain extracts | Involved in cell adhesion during  neurodegeneration; a target for allergic respiratory diseases, including asthma |
| ADAM9 |  | not present overt phenotypes | Involved in induced ectodomain shedding of  membrane-anchored heparin-binding EGF-like  growth factor; suggested to cleave APP at the a-secretase site |
| ADAM10 | Central nervous system function | early embryonic lethality and multiple defects of the developing CNS, somites, and cardiovascular system | Involved in shedding of various transmembrane  proteins, including cadherins Major Notch-site 2  protease; a-secretase for APP; controls wound  healing, neurogenesis, skin homeostasis |
| ADAM11 | Central nervous system function |  | Candidate tumor suppressor gene for human breast cancer, pain transmission, synaptic modulation |
| ADAM12 |  | not present overt phenotypes | Involved in myogenesis and skeletal muscle  regeneration; upregulated in tumor progression;  involved in macrophage-derived giant cell (MGC)  and osteoclast formation from mononuclear  precursors |
| ADAM15 |  | an accelerated development of osteoarthritic lesions in aging mice | Involved in cell adhesion through integrin binding; involved in wound healing; mediates heterotypic  -cell interactions; cleaves E-cadherin in response to growth factor deprivation; plays a role in glomerular cell migration and pathological neovascularization; involved in cartilage remodeling and sperm-egg binding |
| ADAM17 | Central nervous system function | a perinatal lethality with open eye lids, a lack of a conjunctival sac, thinned corneas, and epidermal and hair defects | Cleaves TNFo and is responsible for proteolytic release of several other cell-surface proteins, including p75 TNF receptor, interleukin 1 receptor type II, p55 TNF receptor, TGFo, L-selectin, growth hormone receptor, MUC1 and amyloid precursor protein; possibly involved in the activation of Notch pathway |
| ADAM18 | Fertilization |  | Possibly involved in sperm-egg fusion |
| ADAM19 |  | severe defects in cardiac morphogenesis and abnormalities of the cardiac vasculature | Shedding of beta-type neuregulin isoforms involved in neurogenesis and synaptogenesis; possibly involved in osteoblast differentiation and/or osteoblast activity in bone |
| ADAM20 | Fertilization |  | Possibly involved in sperm-egg fusion; testis-specific expression |
| ADAM21 | Central nervous system function |  | Possibly involved in sperm-egg fusion and in epithelia functions |
| ADAM22 | Central nervous system function | ataxia, convulsions and peripheral neuropathy were observed | Ligand for integrin in the brain; involved in regulation of cell adhesion and in inhibition of cell proliferation; neuronal receptor for LGI1 |
| ADAM 23 | Central nervous system function |  | Involved in cell-cell and cell-matrix interactions in brain |
| ADAM 28 |  |  | Possible role during lymphocyte emigration;  shedding of lymphocyte surface target proteins, such as FASL and CD40L; might be involved in sperm maturation |
| ADAM 29 |  |  | Involved in spermatogenesis and fertilization |
| ADAM 30 |  |  | Involved in spermatogenesis and fertilization |
| ADAM 32 |  |  | Involved in spermatogenesis and fertilization |
| ADAM 33 |  |  | Possibly involved in asthma and bronchial hyper-responsiveness |

ADAM: A Disintegrin and metalloprotease, CHL1: Close Homologue of L1, EGF: Epidermal growth factor, APP: amyloid precursor protein, CNS: Central nervous system, MGC: macrophage-derived giant cell, TNF: Tumor necrosis factor, TGF: Transforming growth factor, LGI1: anti-leucine-rich glioma-inactivated 1, FASL: FAS-Ligand, CD40L: CD40-Ligand.
